# Can Small Hepatocellular Carcinoma be Cured by Percutaneous Acetic Acid Injection Therapy?

**DOI:** 10.1155/1997/73596

**Published:** 1997

**Authors:** S.-T. Fan

**Affiliations:** Department of Surgery The University of Hong Kong Queen Mary Hospital Hong Kong

## Abstract

To assess the efficacy of ultrasound (US)-guided percutaneous acetic acid (in concentrations of 15%, 20%, 30%, 40%, and 50%) injection for small hepatocellular carcinomas (HCCs) for long-term prognosis, percutaneous acetic acid injection using 15% to 50% acetic acid was performed in 91 patients with one to four HCCs smaller than 3 cm during the past 6.5 years. During the series of treatment sessions for each patient, the same concentration of acetic acid was used. All tumors could be treated successfully with percutaneous acetic acid injection despite the differences in acetic acid concentration used. The number of treatment sessions to treat similar size of tumor was less when the higher concentration of acetic acid was used. No serious complications occurred as a direct sequela to percutaneous acetic acid injection. None of the tumor treated regrew. The 1-, 2-, 3-, 4-, and 5-year survival rates for 91 patients were 95%, 87%, 80%, 63%, and 49%, respectively. The 1-, 2-, 3-, 4-, and 5-year cancer-free survival rates of these patients were 83%, 54%, 50%, 37%, and 29%, respectively. Both liver function and size of tumor affected both survival rate and cancer-free survival rate significantly, but the number of tumors did not. The concentration of acetic acid did not affect the survival rate. Percutaneous acetic acid using 15% to 50% acetic acid will be effective therapy for small HCCs for long-term prognosis.


ment either on the day of their operation or one day later. The issue at hand now is to compare laparoscopic cholecystectomy to "small-incision" cholecystectomy in a properly designed trial, in such a setting.

*References*

[1] Barkun, J. S., Barkun, A. N. and Sampalis, J. S. *et al.* and the McGill gallstone treatment group. (1992). Randomised controlled trial of laparoscopic versus mini cholecystectomy, *Lancet*, **340**, 1116–19.
[2] McMahon, A. J., Russel, I. T. and Baxter, J. N. *et al.* (1994). Laparoscopic versus minilaparotomy cholecystectomy: a randomised trial, *Lancet*, **343**, 135–38.
[3] McGinn, F. P., Miles, A. J. G., Uglow, M., Ozmen, M., Terzi, C. and Humby, M. (1995). Randomized trial of laparoscopic cholecystectomy and mini-cholecystectomy, *Br. J. Surg.*, **82**, 1374–77.
[4] McMahon, A. J., Russell, I. T. and Ramsay, G. *et al.* (1994). Laparoscopic and minilaparotomy cholecystectomy: A randomized trial comparing postoperative pain and pulmonary function, *Surgery*, **115**, 533–539.
[5] Wherry, D. C., Rob, C. G., Marohn, M. R. *et al.* (1994). An external audit of laparoscopic cholecystectomy performed in medical treatment facilities of the Department of Defense, *Ann. Surg.*, **220**, 626–634.

Charles J. Yeo, MD
Professor of Surgery
Johns Hopkins University
School of Medicine
Blalock 606
600 N Wolfe Street
Baltimore, MD 21287- 4606
United States of America

### Can Small Hepatocellular Carcinoma be Cured by Percutaneous Acetic Acid Injection Therapy?

### ABSTRACT

*Ohishi, K., Nomura, F., Ito, S. and Fujiwara, K. (1996) Prognosis of small hepatocellular carcinoma (less than 3cm) after percutaneous acetic acid injection: Study of 91 cases. Hepatology; 23, 994–1002.*

**To assess the efficacy of ultrasound (US)-guided percutaneous acetic acid (in concentrations of 15%, 20%, 30%, 40%, and 50%) injection for small hepatocellular carcinomas (HCCs) for long-term prognosis, percutaneous acetic acid injection using 15% to 50% acetic acid was performed in 91 patients with one to four HCCs smaller than 3 cm during the past 6.5 years. During the series of treatment sessions for each patient, the same concentration of acetic acid was used. All tumors could be treated successfully with percutaneous acetic acid injection despite the differences in acetic acid concentration used. The number of treatment sessions to treat similar size of tumor was less when the higher concentration of acetic acid was used. No serious complications occurred as a direct sequela to percutaneous acetic acid injection. None of the tumor treated regrew. The 1-, 2-, 3-, 4-, and 5-year survival rates for 91 patients were 95%, 87%, 80%, 63%, and 49%, respectively. The 1-, 2-, 3-, 4-, and 5-year cancer-free survival rates of these patients were 83%, 54%, 50%, 37%, and 29%, respectively. Both liver function and size of tumor affected both survival rate and cancer-free survival rate significantly, but the number of tumors did not. The concentration of acetic acid did not affect the survival rate. Percutaneous acetic acid using 15% to 50% acetic acid will be effective therapy for small HCCs for long-term prognosis. (Hepatology 1996; 23, 994–1002).**

*Keywords:* Hepatocellular carcinoma, alcohol injection, acetic acid injection

### PAPER DISCUSSION

The study by Ohnishi *et al.* [1] recommended that acetic acid is the preferred agent to absolute alcohol because acetic acid has the property of extracting collagen and probably good penetration into cancer cells in the tumour capsule or intratumour septa. Thus the number of sessions required to completely necrose the hepatocellular carninoma (HCC) was reduced, the incidence of local recurrence was less and the survival was longer compared with patients treated by percutaneous alcohol injection as reported in the literature. The result is commendable, especially for patients with Child's A liver function. However, not mentioned in the report is whether patients with Child's A function had been offered surgical resection since they constituted almost 70% of the series. Although surgical resection was shown in a non-randomized and retrospective study [2] to achieve a comparable result with percutaneous alcohol injection, partial hepatectomy should not be discarded especially for lesions located at the surface of liver. In that situation, the lesion is not suitable for percutaneous alcohol or acetic acid injection whereas partial hepatectomy would be much safer and more efficacious.

Although almost half of the patients treated by percutaneous acetic acid injection survived 5 years, the cancer-free survival rate was only 29%, indicating that recurrence or new growth is the intrinsic problem in treatment of HCC, irrespective of modality. Since all the hepatocytes have been inflicted by the same carcinogenic agent, intrahepatic recurrence would be invariable and a radical treatment could not be possible without liver transplantation. Liver transplantation was shown to produce a much better result than resection if the HCC is <5 cm. [3]. However, in the face of organ shortage, many patients with small HCC have to be treated by non-surgical means even though the liver function is suboptimal. The method suggested by Ohnishi *et al.* [1] is certainly a step forward in the non-operative treatment of HCC and can 'buy' time before an organ is available.

Prevention of intrahepatic recurrence following surgical or non-surgical treatment is the aim of all surgeons and physicians looking after patients with HCC. Adjuvant treatment with chemoembolization was shown to be effective but that was a non-randomized and retrospective study [4]. The authors had not mentioned whether percutaneous acetic acid had been given to new HCC that developed after the initial treatment. I believe that they had done so. However, the crux of the problem does not lie on how good is the treatment method but depends on the hepatocarcinogenic potential of the hepatocytes of the liver remnant. Polyprenoic acid, by its action of promoting apotopsis and, maturation of hepatoma cell line was shown to reduce development of second primary HCC after hepatectomy or percutaneous alcohol injection [5]. If proven to be of value, it would be the drug for patients with treated HCC and even for high risk individuals for the development of HCC.

### *References*

[1] Ohnishi, K., Nomura, F., Ito, S. and Fujiwara, K. (1996). Prognosis of small hepatocellular carcinoma (less than 3 cm) after percutaneous acetic acid injection: study of 91 cases, *Hepatology*, **23**, 994-1002.

[2] Castells, A., Bruix, J. and Bru, C. *et al.* (1993). Treatment of small hepatocellular carcinoma in cirrhotic patients: a cohort study comparing surgical resection and percutaneous ethanol injection, *Hepatology*, **18**, 1121-1126.

[3] Bismuth, H., Chiche, L., Adam, R., Castaing, D., Diamond, T. and Dennison, A. (1993). Liver resection versus transplantation for hepatocellular carcinoma in cirrhotic patients, *Ann. Surg.*, **218**, 145-151.

[4] Nonami, T., Isshiki, K. and Katoh, H. *et al.* (1991). The potential role of postoperative hepatic artery chemotherapy in patients with high-risk hepatomas, *Ann. Surg.*, **213**, 222-226.

[5] Muto, Y., Moriwaki, H. and Ninomiya, M. *et al.* (1996). Prevention of second primary tumors by an acyclic retinoid, polyprenoic acid, in patients with hepatocellular carcinoma, *N. Engl. J. Med.*, **334**, 1561-1567.

Professor S.-T. Fan
Department of Surgery
The University of Hong Kong
Queen Mary Hospital
Hong Kong

